# Evaluation of postoperative pain associated with different working length determination methods: a randomised clinical study

**DOI:** 10.1186/s12903-026-09451-8

**Published:** 2026-08-08

**Authors:** Gamze Nalci Calik, Betül Aycan Alim Uysal, Fatma Begüm Peker, Mehmet Burak Guneser

**Affiliations:** 1 Department of Endodontics, Bezmialem Vakif University, Faculty of Dentistry, Istanbul, Türkiye; 2 Department of Endodontics, Health Science University, Faculty of Dentistry, Istanbul, Türkiye

**Keywords:** Electronic apex locator, Working length, Postoperative pain, Root canal therapy

## Abstract

**Background:**

Postoperative pain is a common concern in endodontic treatment. In teeth with asymptomatic irreversible pulpitis, accurate working length determination is critical for effective root canal therapy and may influence postoperative discomfort. This study compared postoperative pain following root canal treatment in teeth with asymptomatic irreversible pulpitis when working length was determined using one of four electronic apex locators or periapical radiography.

**Methods:**

A total of 125 patients with asymptomatic irreversible pulpitis in single-rooted, single-canal teeth were included in the analysis. Patients were randomly assigned to five groups according to the working length determination method: Root ZX, Propex Pixi, Woodpex III, Raypex 6, or periapical radiography. A single clinician performed standardised root canal treatment in a single visit. Pain was assessed using the visual analogue scale at 6, 12, 24, and 48 hours postoperatively, and analgesic intake was recorded.

**Results:**

Postoperative pain gradually decreased over time in all groups. No statistically significant differences in postoperative pain were observed among the five working length determination methods at any postoperative assessment time point (all *P* > 0.05). Furthermore, postoperative pain was not significantly associated with age, sex, tooth location, and analgesic intake did not differ significantly among the groups.

**Conclusion:**

Under the standardised clinical conditions of this randomised clinical trial, the method used for working length determination did not significantly influence postoperative pain after single-visit root canal treatment in patients with asymptomatic irreversible pulpitis.

**Trial registration:**

This study was retrospectively registered at ClinicalTrials.gov (Identifier: NCT07271797) on November 16, 2025. However, the study protocol, eligibility criteria, primary and secondary outcome measures, and statistical analysis plan were established before patient enrolment.

## Introduction

Working length (WL) is defined as the measured distance from a fixed coronal reference point to the point at which root canal preparation and obturation should be terminated [1]. Accurate determination of the WL is fundamental to the success of endodontic therapy, as it ensures effective instrumentation and obturation while minimising procedural errors and complications [2, 3].

Optimal root canal treatment outcomes are achieved when obturation is completed at the apical constriction, which is considered the narrowest part of the root canal system and the region of minimal vascularisation [4–7]. Therefore, avoiding both over-instrumentation and under-preparation is essential, as either may compromise treatment outcomes. Traditionally, radiography has been the most widely used method for determining WL, allowing the position of an endodontic instrument within the canal to be visualised [8]. However, radiographic assessment has inherent limitations, including its two-dimensional nature and potential inaccuracies in identifying the apical foramen. To address these limitations, electronic apex locators (EALs) have become integral tools in modern endodontic practice, providing a more efficient and precise alternative for WL determination by improving the accuracy of apical localisation [9]. Additionally, EALs have been reported to reduce chair time, improve patient comfort, and simplify clinical workflows, thereby contributing to their widespread adoption [6, 10, 11].

A range of EALs with different technological features and generations are currently available, and these devices differ in their operating principles, including frequency-based measurements and adaptive signal processing, which may influence their performance in working length determination [12–15]. Such variations could potentially affect apical preparation and the extent of periapical irritation, thereby influencing postoperative pain [16]. Therefore, comparing different generations of EALs may provide clinically relevant insights into their influence on postoperative outcomes.

Inaccuracies in working length, such as over-instrumentation or underfilling, may lead to unfavourable outcomes, including post-treatment flare-ups, delayed tissue healing, persistent pain, and treatment failure [17, 18]. Over-instrumentation may result in the extrusion of debris, microorganisms, or irrigants into the periapical tissues, triggering an inflammatory response, whereas under-preparation may leave infected tissue within the canal, contributing to persistent postoperative discomfort [19].

This study aimed to compare postoperative pain following root canal treatment in teeth with asymptomatic irreversible pulpitis, using four different EALs and periapical radiography for working length determination. The null hypothesis was that there would be no statistically significant difference in postoperative pain levels among the working length determination methods.

## Materials and methods

The study was approved by the Bezmialem Vakif University Clinical Research Ethics Committee (28.06.2021-E.21696). All methods were carried out in accordance with relevant guidelines and regulations, including the Declaration of Helsinki. Written informed consent was obtained from all participants prior to enrolment. This clinical trial was retrospectively registered at ClinicalTrials.gov (Identifier: NCT07271797) on November 16, 2025. Although the study was retrospectively registered, the study protocol, inclusion criteria, and outcome measures were predefined prior to patient enrolment and remained unchanged after data collection.

An a priori sample size calculation was performed using G*Power software (version 3.1.9.7; Heinrich Heine University Düsseldorf, Düsseldorf, Germany). The calculation was based on the primary outcome (postoperative pain at 6 hours) using the between-group differences reported by Guzel et al. A one-way fixed-effects omnibus ANOVA model with five independent groups was used [20]. Assuming an effect size of f = 0.400, an alpha level of 0.05, and a statistical power of 95%, the minimum required sample size was calculated to be 125 participants (25 per group). Accordingly, 125 patients aged 18–60 years diagnosed with asymptomatic irreversible pulpitis at the Department of Endodontics, Faculty of Dentistry, Bezmialem Vakif University, were included in the present study.

Patients with asymptomatic irreversible pulpitis were eligible if they met all of the following criteria: no history of systemic conditions that could affect pain perception or healing (e.g., immunocompromised status, uncontrolled systemic disease, and bleeding disorders); vital single-rooted teeth with a single root canal and no apical resorption; no intake of analgesics in the 12 hours preceding root canal treatment; no sensitivity to percussion or palpation; and not being pregnant at the time of treatment. The diagnosis of asymptomatic irreversible pulpitis was established on the basis of clinical and radiographic criteria, including a positive response to cold testing (prolonged pain), the absence of spontaneous pain, the absence of percussion or palpation sensitivity, and no evidence of periapical radiolucency on preoperative radiographs. Patients with calcified root canals, root resorption, periodontal problems, a history of previous endodontic treatment, or a periapical lesion on the diagnostic radiograph were excluded. Written consent was obtained from all participants.

Preoperative periapical radiographs were taken using the paralleling technique under standard exposure conditions. Age, sex, tooth type, and VAS results were recorded for all participants, who were not given any additional information about which method was used in their treatment. A single endodontist performed the entire endodontic procedure for all patients using a standardised protocol in a single visit. Rubber dam isolation was used in every case, and local anaesthesia (Articaine 4% with 1:200,000 epinephrine, Ultracaine DS Fort, Hoechst-Marion Roussel, Frankfurt, Germany) was administered. A standard access cavity was prepared using sterile diamond and carbide burs.

Participants were assigned to the five study groups using simple randomisation with a computer-generated random allocation sequence prepared by a second researcher who was not involved in the treatment procedures. The treatment allocation was communicated to the operator only after participant enrolment, thereby maintaining allocation concealment until assignment. Operator blinding, however, was not feasible because the clinician could readily identify the electronic apex locator used during treatment. The study groups were as follows.

- Group 1: Root ZX (Morita Corp., Tokyo, Japan)
- Group 2: Propex Pixi (Dentsply Maillefer, Ballaigues, Switzerland)
- Group 3: Woodpex III (Woodpecker Medical Instrument Co., Guilin, China)
- Group 4: Raypex 6 (VDW, Munich, Germany)
- Group 5: Periapical radiography

Canal patency was established using a sterile ISO size 15 stainless-steel K-file (Mani, Japan). All devices in Groups 1–4 were used in accordance with the manufacturers’ recommendations, and the WL in the EAL groups was determined accordingly. In the radiographic evaluation group, a size-15 K-file (Mani, Japan) was inserted into the root canal beyond the estimated WL, and a periapical radiograph was then obtained using a phosphor plate and the paralleling technique (DÜRR Dental GmbH & Co., Bietigheim-Bissingen, Germany). When the file tip extended beyond or fell short of the apex, the WL was recalculated, and the final WL was set 1 mm short of the radiographic apex.

All canals were prepared using the VDW ROTATE NiTi system (VDW, Munich, Germany) with an endodontic motor (X-Smart Plus, Dentsply Maillefer) to a standardised apical size of #40 with a 0.04 taper. This preparation size was selected because it is commonly used for single-canal teeth and provides adequate canal enlargement for irrigation and debridement while maintaining procedural standardisation across all study groups. The same preparation protocol was applied to all canals to minimise variability related to instrumentation. Although a standardised protocol was applied, anatomical variations inherent to in vivo conditions may limit complete standardisation.

Irrigation was performed using 2.5% sodium hypochlorite delivered with a 30-gauge side-vented needle, with approximately 2 mL of irrigant was used after each instrument. Following instrumentation, 2 mL of 17% EDTA was applied for smear layer removal for 1 minute, followed by a final rinse with saline to eliminate residual chelating effects. The canals were obturated by lateral compaction using a resin-based sealer (ADSEAL; Meta Biomed Co., Ltd., Cheongju, Republic of Korea).

**Fig. 1:**
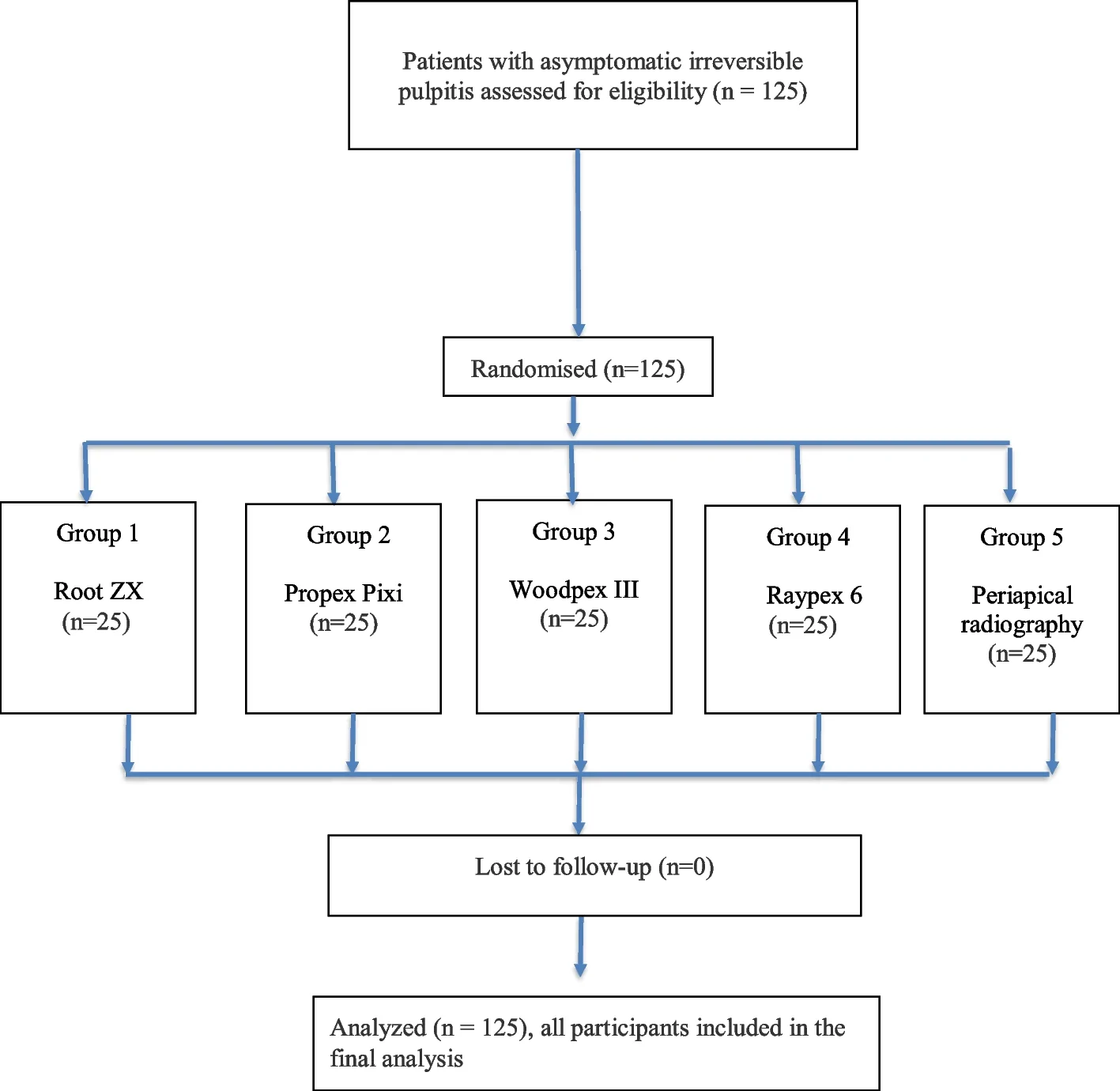
CONSORT flow diagram of the study

Postoperative pain was assessed using a patient-completed visual analogue scale (VAS) diary. A 10-cm line ranging from ‘no pain’ to ‘worst possible pain’ was used, and the distance from the ‘no pain’ end to the marked point was recorded as the pain score. Participants were instructed in the use of the VAS before the assessment and were asked to record their pain scores at 6, 12, 24, and 48 hours after treatment at home. The completed forms were subsequently collected by a third researcher, who was not involved in the randomisation or treatment procedures, via telephone contact and photographic documentation, thereby ensuring partial blinding of outcome assessment and reducing the risk of observer bias. All participants followed the same data collection procedure. Analgesics (400 mg ibuprofen) were prescribed at 6-hour intervals if pain was severe, and the patients were asked to record the number of analgesics used per day. The study workflow is shown in Fig. 1.

### Statistical analysis

Data were analysed using R software (version 4.5). As postoperative pain scores were not normally distributed, comparisons among the five working length determination groups at each assessment time point were performed using the Kruskal–Wallis H test. Comparisons between male and female participants were performed using the Mann–Whitney U test, and associations between age and postoperative pain scores were evaluated using Spearman’s rank correlation coefficient (Spearman’s rho). Continuous variables are presented as median (minimum–maximum) together with mean ranks, whereas categorical variables are presented as frequencies and percentages. Effect sizes for the Kruskal–Wallis H test were calculated as epsilon squared (ε2) using the H/(N−1) estimator, whereas effect sizes for the Mann–Whitney U test were expressed as Z-based Cohen's d equivalents with 95% confidence intervals. All statistical tests were two-sided, and *P* < 0.05 was considered statistically significant.

## Results

A total of 125 patients with asymptomatic irreversible pulpitis completed the study (62 males, 49.6%; 63 females, 50.4%). Participants ranged in age from 18 to 60 years.

**Fig. 2:**
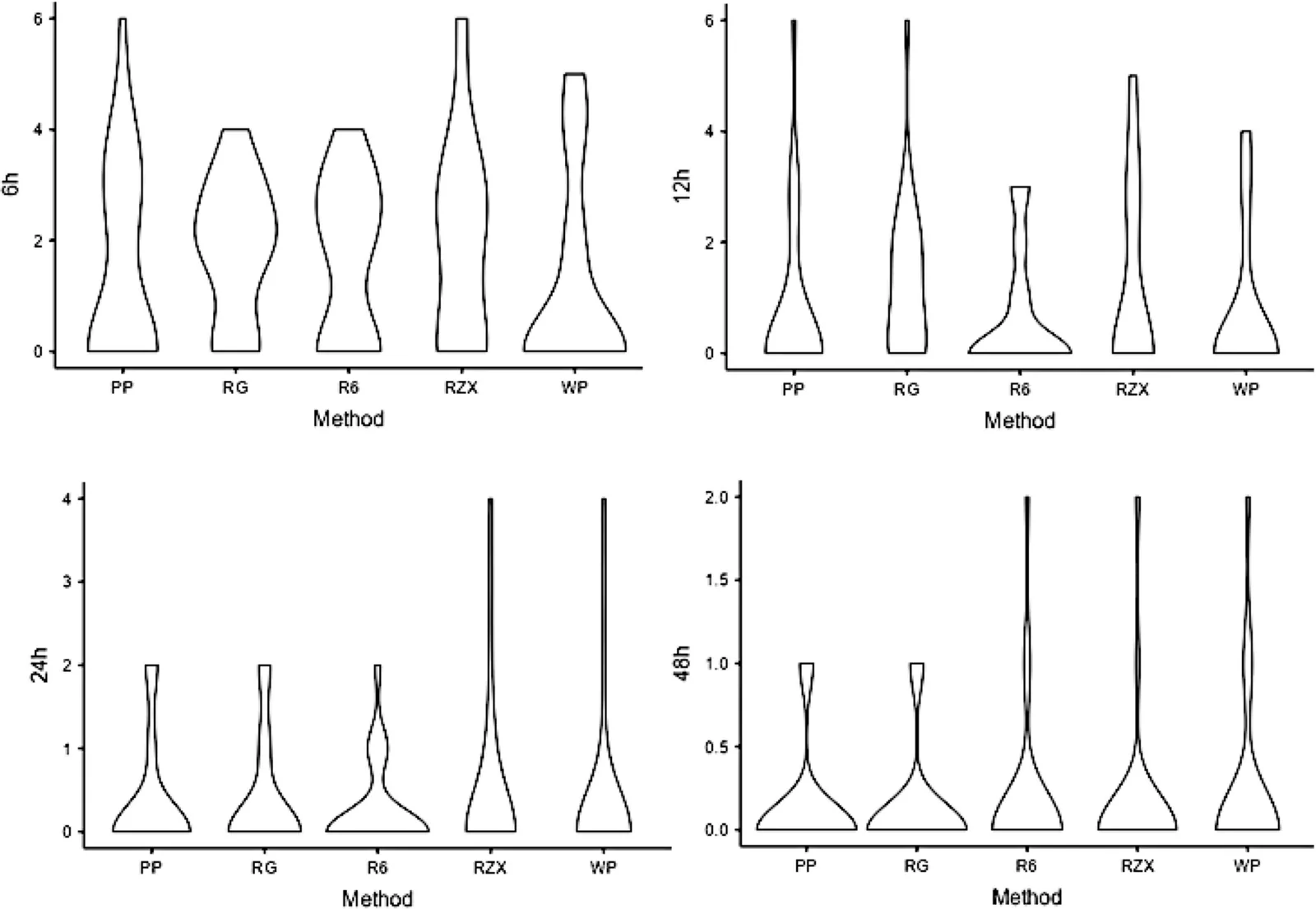
Violin plots showing the distribution of postoperative pain scores at 6, 12, 24, and 48 hours (h) according to the working length determination method. WP: Woodpex III; PP: Propex Pixi; R6: Raypex 6; RZX: Root ZX; RG: Radiographic method

Postoperative pain scores followed a consistent downward trend from 6 to 48 hours across all study groups (Fig. 2). Intergroup comparisons revealed no statistically significant differences in pain scores at any time point (6 h: *P* = 0.363; 12 h: *P* = 0.174; 24 h: *P* = 0.966; 48 h: *P* = 0.949; Table 1). The effect sizes (ϵ^2^) for these comparisons were small (0.005–0.051), suggesting that the influence of the working length determination method on postoperative discomfort was minimal.

**Table 1:** Comparison of postoperative pain scores at each assessment time point according to the working length determination method

Time (hours)	PP	RG	R6	RZX	WP	Total	Test statistic	*P* value	Effect size (ε^2^)
6	0 (0: 6)/60	2 (0: 4)/69	2 (0: 4)/64	2 (0: 6)/69	0 (0: 5)/52	2 (0: 6)	4.333	0.363	0.035
12	0 (0: 6)/57	1 (0: 6)/75	0 (0: 3)/59	0 (0: 5)/67	0 (0: 4)/57	0 (0: 6)	6.365	0.174	0.051
24	0 (0: 2)/63	0 (0: 2)/65	0 (0: 2)/62	0 (0: 4)/65	0 (0: 4)/61	0 (0: 4)	0.578	0.966	0.005
48	0 (0: 1)/63	0 (0: 1)/63	0 (0: 2)/63	0 (0: 2)/61	0 (0: 2)/66	0 (0: 2)	0.721	0.949	0.006

Data are presented as median (minimum–maximum); values following the slash represent mean ranks. Comparisons among groups were performed using the Kruskal–Wallis H test. Effect sizes are expressed as epsilon squared (ε^2^) using the H/(N−1) estimator. WP: Woodpex III; PP: Propex Pixi; R6: Raypex 6; RZX: Root ZX; RG: Radiographic method

Regarding secondary variables, no statistically significant differences in postoperative pain scores were observed between male and female participants at any assessment time point (*P* > 0.05; Table 2). Similarly, no significant correlations were found between age and postoperative pain scores (*P* > 0.05; Table 3). Furthermore, tooth location (mandible [44%] vs. maxilla [56%]) had no significant effect on postoperative pain (*P* > 0.05).

**Table 2:** Comparison of postoperative pain scores between male and female participants at each assessment time point

Time (hours)	Male	Female	Total	Test statistic	*P* value	Effect size (Cohen’s d)
6	2 (0: 6)/63.73	2 (0: 6)/62.28	2 (0: 6)	−0.225	0.822	0.040 [−0.311, 0.391]
12	0 (0: 6)/65.42	0 (0: 6)/60.62	0 (0: 6)	−0.741	0.459	0.133 [−0.219, 0.484]
24	0 (0: 3)/61	0 (0: 4)/64.97	0 (0: 4)	−0.612	0.540	0.110 [−0.241, 0.460]
48	0 (0: 1)/59.44	0 (0: 2)/66.51	0 (0: 2)	−1.091	0.275	0.196 [−0.156, 0.547]

Data are presented as median (minimum–maximum); values following the slash represent mean ranks. Comparisons between male and female participants were performed using the Mann–Whitney U test. Effect sizes are reported as Cohen's d equivalents derived from the Z statistic (η2-based transformation), with 95% confidence intervals

**Table 3:** Correlation between age and postoperative pain scores at each assessment time point

Time (hours)	Spearman's rho (r)	*P* value
6	0.002	0.985
12	−0.087	0.334
24	−0.082	0.361
48	0.044	0.625

rho, Spearman’s rank correlation coefficient

Analgesic intake did not differ significantly among the study groups (*P* > 0.05). Overall, 21 patients reported analgesic intake at 6 hours, 16 at 12 hours, 7 at 24 hours, and 2 at 48 hours.

## Discussion

Accurate WL determination is essential for successful root canal treatment, as deviations may result in over-instrumentation or insufficient debridement of the root canal system [6, 21–23]. The European Society of Endodontology recommends the combined use of electronic and radiographic methods for WL determination [24]. This study aimed to evaluate whether different WL determination methods influence postoperative pain following single-visit root canal treatment.

The principal finding of this study was that the method used for WL determination, whether different generations of EALs or periapical radiography, did not significantly influence postoperative pain intensity. Accordingly, the null hypothesis was not rejected. These findings are consistent with those of a recent systematic review by Sultan et al., which concluded that standalone EALs and radiographic methods are associated with comparable postoperative pain outcomes following single-visit root canal treatment [4]. Furthermore, the small effect sizes (ε^2^ = 0.005–0.051) observed across all postoperative assessment time points suggest that any numerical differences between groups were unlikely to be clinically meaningful. This interpretation is further supported by the consistently low median pain scores observed across all study groups. Although the evaluated devices differ in their operating principles and technological generations, modern EALs are all designed to identify the apical constriction with a high degree of accuracy. Therefore, any technical differences among the devices may not be sufficiently large to produce clinically detectable differences in postoperative pain.

Previous studies have demonstrated high accuracy rates for various EALs, particularly Root ZX [25–27]. However, this study was not designed to evaluate the accuracy of the tested devices; rather, it investigated whether different WL determination methods influenced postoperative pain. The absence of significant differences among the evaluated methods suggests that variations in device characteristics, technological generation, or the use of electronic versus radiographic WL determination may not necessarily translate into differences in patient discomfort. One plausible explanation for these findings is the use of a highly standardised treatment protocol across all study groups. All groups underwent the same instrumentation, irrigation, smear-layer removal, and obturation protocols. A uniform apical preparation size of 40/.04 was used to promote adequate debridement of the apical third while reducing procedural variability among the groups. Consequently, procedural variability unrelated to working length determination was minimised, and this standardisation may have contributed to the comparable postoperative pain outcomes observed across the groups.

Postoperative endodontic pain is a multifactorial outcome influenced by several patient- and treatment-related factors, including age, sex, pulpal status, and type of treatment performed (primary root canal treatment or retreatment) [28]. To minimise the influence of treatment-related confounding factors, all root canal treatments in the present study were completed in a single visit. This approach was adopted because previous studies have demonstrated that single-visit root canal treatment results in postoperative pain and periapical healing outcomes similar to those of multiple-visit treatment while eliminating the potential variability associated with intracanal medicaments [29, 30].

Previous studies have reported inconsistent findings regarding the influence of sex on postoperative endodontic pain, with some authors suggesting greater pain perception among female patients because of hormonal and physiological factors [31–33]. In contrast, no significant sex-related differences were observed in this study, and the small effect sizes (Cohen's *d* = 0.040–0.196) also indicated that sex had little influence on postoperative discomfort. These findings are consistent with recent endodontic studies that reported no clinically meaningful association between patient sex and postoperative pain under comparable clinical conditions [18–20]. A possible explanation is that all participants presented with the same pulpal diagnosis and generally experienced low levels of postoperative pain, thereby reducing the likelihood that sex-related differences in pain perception would become clinically apparent.

Although age-related anatomical changes have been suggested to complicate working length determination, particularly because of increased cementum deposition and alterations in the relationship between the apical constriction and apical foramen, these changes did not appear to influence postoperative pain in the present study [5]. This observation is consistent with previous clinical studies conducted in both paediatric and adult populations, which likewise reported no significant association between patient age and postoperative pain following endodontic treatment [18–20]. Taken together, these findings suggest that although ageing may influence root canal anatomy, its impact on postoperative pain appears to be limited.

Regardless of the working length determination method, postoperative pain was highest at 6 hours and gradually decreased thereafter, with minimal pain reported by 48 hours. This temporal pattern is consistent with previous studies [25–27]. The initial peak may reflect the waning effect of local anaesthesia together with the onset of an acute inflammatory response in the periapical tissues following instrumentation, whereas the subsequent decline in pain by 48 hours is likely attributable to the resolution of acute inflammation and early tissue adaptation. The consistent reduction in pain across all groups further suggests that postoperative healing followed a similar clinical course, regardless of the WL determination method used.

This study has several limitations. First, operator blinding was not feasible due to the nature of the intervention, which may have introduced performance bias. Second, all procedures were performed by a single operator, which ensured procedural standardisation but may limit the generalisability of the findings. Although a standardised apical preparation protocol was applied, complete standardisation may be inherently limited under in vivo conditions due to anatomical variability. In addition, the follow-up period was limited to 48 hours, as postoperative pain is known to peak within the early period after treatment; however, a longer follow-up period would provide additional insight into the progression of postoperative pain. Furthermore, the accuracy of the EALs was not assessed in the present study, and no conclusions can therefore be drawn regarding the relationship between device accuracy and postoperative pain. Finally, although potential confounding variables such as age, sex, and tooth location were examined individually, residual confounding from unmeasured factors, including detailed tooth type and root canal morphology, cannot be excluded.

Despite these limitations, this study has several strengths. A standardised treatment protocol was applied to all patients, and all procedures were performed by a single operator, ensuring procedural consistency. The sample size was adequate and supported by a power analysis, and postoperative pain was evaluated at multiple time points, allowing a detailed assessment of pain progression. In addition, the inclusion of multiple generations of EALs together with a radiographic comparison group provided a comprehensive evaluation of working length determination methods commonly used in contemporary clinical practice.

## Conclusion

No significant differences in postoperative pain were observed among the evaluated working length determination methods. Therefore, the choice of working length determination method appears unlikely to influence postoperative pain following single-visit root canal treatment in teeth with asymptomatic irreversible pulpitis.

## Data Availability

The datasets generated and/or analysed during the current study are available from the corresponding author upon reasonable request.
